# Discovering Drugs for the Treatment of Ebola Virus

**DOI:** 10.1007/s40506-017-0130-z

**Published:** 2017-08-04

**Authors:** Sandra L. Bixler, Allen J. Duplantier, Sina Bavari

**Affiliations:** United States Army Medical Research Institute of Infectious Diseases, 1425 Porter St, Frederick, MD 21702 USA

**Keywords:** Ebola virus, Viral hemorrhagic fever, Therapeutic, Pharmacokinetic

## Abstract

**Purpose of review:**

Ebola virus, a member of the Filoviridae family, is a causative agent of severe viral hemorrhagic fever in humans. Over the past 40 years, the virus has been linked to several high mortality outbreaks in Africa with the recent West African outbreak resulting in over 11,000 deaths. This review provides a summary of the status of the drug discovery and development process for therapeutics for Ebola virus disease, with a focus on the strategies being used and the challenges facing each stage of the process.

**Recent findings:**

Despite the wealth of in vitro efficacy data, preclinical data in animal models, and human clinical data, no therapeutics have been approved for the treatment of Ebola virus disease. However, several promising candidates, such as ZMapp and GS-5734, have advanced into ongoing clinical trials.

**Summary:**

The gravity of the 2014-2016 outbreak spurred a heightened effort to identify and develop new treatments for Ebola virus disease, including small molecules, immunotherapeutics, host factors, and clinical disease management options.

**Disclaimer:**

Opinions, interpretations, conclusions, and recommendations are those of the authors and are not necessarily endoresed by the U.S. Army.

## Introduction

Drug development for Ebola virus (EBOV) has been in progress for several decades, primarily fueled by concerns about the potential use of the Category A agent as a bioweapon. However, the unprecedented magnitude and scale of the 2014–2016 outbreak in West Africa, combined with the potential spread to other corners of the world, led to a renewed focus on medical countermeasures for Ebola virus disease (EVD).

Ebola virus is a member of the *Filoviridae* family which includes the three genera *Ebolavirus*, *Marburgvirus* and *Cuevavirus* [1]. The genus *Ebolavirus* contains its eponymous member EBOV, in addition to four related species: Sudan virus, Tai Forest virus, Bundibugyo virus, and Reston virus [1]. Filoviruses are pleomorphic in shape and are encased in a lipid envelope. The negative-sense single-stranded RNA genome is approximately 19 kb in size, and consists of a linear, non-segmented RNA. The linear viral genome encodes for seven proteins: nucleoprotein (NP), polymerase cofactor VP35, matrix proteins VP40 and VP24, glycoprotein (GP), transcription activator VP30, and RNA-dependent RNA polymerase (L).

Humans are thought to be an “accidental” host for filoviruses as opposed to a natural reservoir, due to the high mortality rates associated with EVD outbreaks in humans. Serological data indicates that bats are likely a natural reservoir, or other species yet to be identified [2]. Transmission occurs through contact with bodily fluids of an infected patient or animal, either through direct inoculation such as a needlestick or exposure of broken skin and/or mucous membranes. While early cellular targets for infection are dendritic cells, monocytes, and macrophages, a variety of cells are infected by EBOV as the disease progresses [3]. EBOV infection of cells leads to dysregulation of the immune response including suppression of type I interferon responses due to the action of viral proteins such as VP24 and VP35 [4•]. Conversely, massive release of proinflammatory cytokines and chemokines, termed “cytokine storm,” is also a hallmark of EBOV infection and likely contributes to inflammation and other disease manifestations [4•, 5].

Ebola virus is a causative agent of viral hemorrhagic fever which is associated with mortality rates as high as 90% in humans [6, 7]. Initial clinical signs of EVD include non-specific symptoms such as fever, malaise, and gastrointestinal involvement, followed by a rapid progression to shock, organ failure, and death [8•]. Despite being a causative agent of viral hemorrhagic fever, hemorrhage is often only present in a fraction of cases. Some patients may present with petechiae and/or a maculopapular rash. Common laboratory findings in EVD include lymphopenia, anemia, elevated liver enzymes, and evidence of coagulopathy including thrombocytopenia and high levels of D-dimers [8•].

Although past EVD outbreaks have relied extensively on supportive care measures such as fluid and electrolyte replacement, a number of experimental therapeutics were evaluated in clinical trials and under compassionate use protocols during the 2014–2016 outbreak. This review focuses on the status of EVD therapeutics at the three main stages of the drug development pathway (discovery, preclinical, and clinical) with a view towards the fundamental principles of pharmacokinetic/pharmacodynamic (PK/PD) relationships such as (a) interaction of the drug with the target, (b) drug exposure at the site of action, and (c) expression of pharmacological activity in the target tissue [9]. This article does not include a discussion of vaccine development efforts and candidates, which have been extensively discussed in other reviews [10].

## Discovery of Compounds for the Treatment of Ebola Virus: Early Steps

### Biological Targets for the Treatment of EVD

Therapeutic development requires the identification of proteins, RNA, or other biological components that will make suitable drug targets. Drug targets, which are identified and validated through a combination of biochemical, genetic, structural, and computational strategies, are generally derived from either the host or pathogen. As such, therapeutic strategies to fight EBOV fall into four main categories: direct targeting of the virus, modulation of host factors, modulation of the immune response, and management of clinical disease.

One of the most popular strategies for EBOV therapeutics is antivirals that directly target critical stages in the viral life cycle such as binding and/or entry of the virus into host cells, viral replication, packaging, or release of viral progeny from target cells. Small molecules, antisense therapies, and immunotherapeutics comprise the diverse list of EBOV antiviral compounds. A disproportionate number of the most advanced therapeutics currently under evaluation are small molecules directed against the RNA-dependent RNA polymerase L required for viral replication. These include the nucleoside analogs BCX4430 [11, 12], GS-5734 [13••], and favipiravir (T-705) [14, 15] which are intracellularly converted to the active nucleoside triphosphate (or nucleotide). The two primary classes of antisense therapies are small-interfering RNAs (siRNAs), which promote degradation of mRNA transcripts, and phosphorodiamidate morpholino oligomers (PMOs) that interfere with translation [16]. TKM-100802 (TKM-Ebola) and its derivative TKM-130803, which was designed for improved targeting of the West African strain of EBOV, are combinations of three siRNAs that hit multiple viral targets (L, VP35, and VP24). AVI-7537 and AVI-6002 (a combination that includes AVI-7537 and AVI-7539) are PMOs that target VP24 and VP24/VP35, respectively. In vitro investigation of other viral proteins such as VP35, VP24, and VP40 as potential new targets for EBOV drugs is currently underway. Direct antivirals also include many of the immunotherapeutics under development that bind to the virus and prevent entry. As the only surface expressed protein of EBOV, GP is a common target of such therapeutic antibodies including the ZMapp antibody cocktail, monoclonal, and polyclonal antibodies. The ZMapp cocktail is comprised of three monoclonal chimeric antibodies with neutralizing activity that target the GP base and glycan cap [17]. Other immunotherapeutics that target GP include lectins such as mannose binding lectin (MBL) which have shown efficacy in in vitro and rodent models [18].

Host factor modulators have gained recent interest in the EBOV field. Like many other viruses with limited genomes, EBOV utilizes host proteins to gain entry and undergo replication. Several host proteins involved in EBOV entry have been tested, including cathepsins, Niemann-Pick C1 (NPC1), and T-cell immunoglobulin and mucin 1 (TIM-1). Cathepsins such as CatB and CatL are cysteine proteases in the endosome that cleave EBOV GP prior to fusion and entry. Although protease and cathepsin inhibitors have shown efficacy in vitro against EBOV [19, 20], it remains unclear whether specific targeting of cathepsins could be used therapeutically due to potential compensatory mechanisms. The cholesterol transport protein Niemann-Pick C1 protein (NPC1) has been shown to bind GP following cathepsin-mediated cleavage. Two small molecules, MBX2254 and MBX2270, are thought to inhibit binding of EBOV GP to NPC1, thus inhibiting infection in in vitro assays [21]. TIM-1 has been shown to bind to GP, thus serving as a receptor for EBOV and other filoviruses [22]. Inhibition of EBOV infection occurred following treatment of cells with the TIM-1 antibody ARD5, suggesting that TIM-1 may be a worthwhile target for EBOV therapeutics [22].

Modulation of the immune system is another strategy that has been investigated for the treatment of EVD. Immunomodulators for EBOV infection, including cytokines, chemokines, and other proteins, may enhance the immune response, thereby promoting viral clearance. They may alternatively dampen undesirable immune responses such as the overwhelming inflammatory cytokine release associated with EBOV. As several EBOV proteins are known to subvert the interferon response, treatment with type I interferons (−α and -β) have been investigated as potential EBOV therapies. The combination of adenovirus-vectored interferon-α with ZMab antibody has been investigated in post-exposure studies in NHPs with success in improving survival and reducing viral loads [23]. Similar treatment of NHPs with interferon-β resulted in extended time to death [24].

Management therapies aim to treat the clinical manifestations of EVD which include coagulation abnormalities and hemorrhagic manifestations. Anticoagulants such as recombinant human activated protein C (rhAPC) and recombinant nematode anticoagulant protein c2 (rNAPc2) that affect the coagulation pathway have been investigated [25, 26]. FX06, a fibrin-derived peptide under development to treat vascular leak syndrome, was administered to patient during the outbreak in an effort to stem vascular leakage [27].

### In Vitro Efficacy Data for EBOV Therapeutics (*Interaction of the Drug With the Target*)

Hit identification for new EBOV therapeutics has occurred mainly through in vitro screening with cell-culture based assays, many of which have high-throughput capability to facilitate the screening of large compound libraries. Those most commonly used are pseudotyped-virus assays that can be performed in biosafety level (BSL)-2 facilities, and replication assays with infectious EBOV which are limited to BSL-4 laboratories. In addition to overcoming biosafety restrictions, pseudotyped-virus assays, in which EBOV GP is expressed on a viral backbone such as HIV or VSV, are useful in determining if the drug inhibits the viral entry process. This information may assist with narrowing down the target or mechanism of action for compounds identified in phenotypic screens. The readouts for these assays include reduction in cytopathic effects or reduction in viral replication as measured by PCR or fluorescent imaging. Table 1 provides a list of compounds and their level of efficacy, reported as either IC_50_ or EC_50_, against EBOV in in vitro assays, although strictly speaking these data all appear to be concentrations of drug that give a half-maximal response.

**Table 1:** In vitro and in vivo efficacy results against EBOV Zaire

Compound/drug	EBOV target	In vitro efficacy data	Animal efficacy data	PK and tolerability	Additional information and comments
BCX4430	Viral polymerase	Kikwit EC_50_: 11.8 μM; EC_90_: 25.4 μM [11]	Mice: 150 mg/kg BID PO; 90% survival; 150 mg/kg BID IM; 100% survival [11] NHP: 25 mg/kg BID D0-14; 100% survival [28]; 100 mg/kg BID IM D2, then 25 mg/kg BID D3-11, 100% survival (67% with same regimen starting on D3) [29]	Well tolerated in all efficacy studies. Very short plasma half-life in mouse and NHP (*T*_1/2_ = 2–10 min). Mouse, 150 mg/kg, IM: liver, *C*_max_ (triphosphate) =65 μM, *T*_max_ = 8 h, *T*_1/2_ = 4.3 h. Conversion to triphosphate in hepatocytes: mouse > human ~ NHP. [11]	BCX4430-TP levels in mouse liver at 150 mg/kg IM are ~×2.5 above EC_90_ value. Distribution into other tissues/cells not reported.
GS-5734	Viral polymerase	Replication: EC_50_: 0.086–0.14 μM [13••]	NHP: 10 mg/kg IV D3-15; 100% protection [13••]	NHP PK 10 mg/kg, IV, short plasma *T*_1/2_ of GS-5734, rapid intracellular conversion to triphosphate with persistent levels >EBOV EC_50_ for 24 h, intracellular triphosphate *T*_1/2_ = 14 h [13••]	NHP tissue distribution plasma ~ testes > eye > brain [13••]
T-705	Viral polymerase	IC_50_: 67 μM [30]	Mice: 100% survival at 300 mg/kg D0-7 against aerosol challenge [31]; 100% survival at 300 mg/kg beginning D6 following intranasal challenge [30]	N/A	Unusually large dose required for efficacy. Intracellular ribosylation required prior to triphosphorylation to active drug
Amodiaquine	Cationic amphiphilic drug (CAD) [32•]	EC_50_: 2.6 μM [33•]/4.4 μM [34] entry EC_50_: 8.4 μM [33•]/34 μM [35] replication *desethyl metabolite has similar EC_50_ value	Mice: no effect at 60 mg/kg BID IP D0–7 [35]	Mouse PK not reported, but differences in male vs. female metabolism noted [36]. Human PK at approved dose of 600 mg PO, *C*_max_ = 87 nM, *T*_1/2_ = 15 h, desethyl-amodiaquine major active metabolite *C*_max_ = 1 ~ 7.6 μM, *T*_1/2_ = 286 h. PPB of both ~90%.	Anti-malarial, N-methyltransferase inhibitor. Free plasma conc of drug and/or active metabolite are both below Ebola EC_50_ at highest approved dose in humans. Drug conc. in tissue may exceed plasma due to high Vd.
Chloroquine	CAD	EC_50_: 4.7 μM entry EC_50_: 16 μM replication [33•]	Mice: mixed results across several dose/studies IP and PO [33•, 35, 37]; Hamsters: no efficacy at 50 mg/kg IP in combination with doxycycline (2.5 mg/kg) and azithromycin (50 mg/kg) [37] GP: 100 mg/kg; no efficacy [38]	Mouse and hamster toxicity (death) at 90 mg/kg, IP [37]. Mouse PK 45 mg/kg IP q12 h: *C*ss = 7.8 μM [33•]. Human PK at 1500 mg PO: *C*_max_ = 3.4 μM, *T*_1/2_ = 13 d [39]. Human PPB =60% [40], mouse not reported.	Anti-malarial. Total drug plasma conc. in mouse (45 mg/kg IP q12hrs) slightly exceeds EBOV EC_50_, but human free drug conc. at 1500 mg falls short. Drug conc. in tissue may exceed plasma due to high Vd
Hydroxychloroquine	CAD	EC_50_: 9.5 μM entry EC_50_: 22 μM replication [33•]	N/A	N/A	Anti-malarial
Aminoquinoline-13	CAD	EC_50_: 4.3 μM entry EC_50_: 21 μM replication [33•]	N/A	Human PK at 1750 mg PO: *C*_max_ = 3.0 μM, *T*_1/2_ = 13 d	Anti-malarial, human total drug conc at 1750 mg below Ebola EC_50_. Drug conc. in tissue may exceed plasma due to high *V*_*d*_
Clomiphene	CAD; entry inhibitor	IC_50_: 11.1 μM (Kikwit) IC_50_: 3.83 μM (Mayinga) [41]	Mice: 60 mg/kg IP QD on days 0, 1, 3, 5, 7, 9; 90% survival [41]	Mouse PK unavailable. Human PK 50 mg QD: *C*_max_ = 37 nM [42]. Protein binding not reported, but likely high due to structure similarity to toremiphene.	Estrogen receptor modulator, human free drug exposure << Ebola EC_50_
Toremiphene	CAD; Entry inhibitor	IC_50_: 1.73 μM (Kikwit) IC_50_: 0.973 μM (Mayinga) [41]	Mice: 60 mg/kg IP QD on days 0, 1, 3, 5, 7, 9; 50% survival [41]	Mouse PK 60 mg/kg PO: C4hr following 3w daily dosing = 0.74 μM [43]. Human PK 120 mg QD: *C*_max_ = 1.8 μM; human PPB =99.5% [44]	Estrogen receptor modulator, Human free drug exposure << Ebola EC_50_. N-desmethyl and 4-hydroxy metabolites are significant.
Amiodarone	CAD, Entry inhibitor	Entry (VSV) IC_50_: 5.6 μM [45] Entry (lentivirus) IC_50_: 2.2 μM [46] Replication IC_50_: 0.4 μM [46]	Mice: 90 mg/kg; 0–40% survival [35]	Human PK variable, ~300 mg/day. *C*_ss_ ~ 3 μM, Vd ~ 60 L/kg [47, 48]; human PPB >96%.	Anti-arrhythmic, human free drug exposure << Ebola EC_50_. Desethyl metabolite significant.
Azithromycin	CAD	EC_50_: 2.79 μM [35]	Mice: 100 mg/kg BID IP; 10–60% survival; no efficacy by PO route GP: no efficacy [35]	Mouse PK 50 mg/kg PO: *C*_max_ = 2.2 μM [49]. Human PK 500 mg PO QD: *C*_max_ = 0.55 μM; human PPB 10–50%	Antibiotic, drug conc in tissue >> plasma
Sertraline	CAD; entry inhibitor	IC_50_: 3.13 μM (Vero) IC_50_: 1.44 μM (HepG2) [50]	Mice: 10 mg/kg; 70% survival [50]	Mouse PK 10 mg/kg IP: *C*_max_ = 1 μM [51]; human PK at approved dose (100–200 mg PO): *C*_max_ = 0.5 μM. PPB 98.5%	SSRI, human free drug plasma exposure << Ebola EC_50_, CNS drug with high brain concentrations
Bepridil	CAD; entry inhibitor	IC_50_: 5.08 μM (Vero) IC_50_: 3.21 μM (HepG2) [50]	Mice: 12 mg/kg; 100% survival [50]	Mouse PK unavailable; Human PK 300 mg PO QD: C_max_ ~ 6.3 μM, PPB >99%	Calcium channel blocker, Human free drug plasma exposure << Ebola EC_50_, QT prolongation issues
Mannose binding lectin	Glycoprotein	Not available	Mice: 350 μg BID Days 0–9; 40% survival; no survival at 75 μg [18]	N/A	N/A
Brincidofovir	Unknown	EC_50_: 120 nm-1.3 μM [52]	No preclinical efficacy reported [53•]	N/A	Interferes with viral DNA replication
Type I IFN	N/A	IFN-α IC_50_: 0.038 μM IFN-β IC_50_: 0.016 μM [54]	IFN-α2b in NHP: delayed time to death [55] IFN-β in NHP: 10.5 μg/kg at 18 h and days 1, 3, 5, 7, 9; delayed time to death [24]	N/A	N/A
TKM-100802/TKM-130803	L, VP35, VP24	Not available	NHP: 100% survival against Kikwit [56••] and Makona [57]	N/A	Lipid nanoparticle formulation of siRNAs
ZMapp	Glycoprotein	N/A	NHP: 100% survival with treatment initiated at D5 [58]	N/A	Antibodies
rhAPC	N/A	Not available	NHP: 18% survival; extended time to death in subset of nonsurvivors [26]	N/A	Protein; treatment of sepsis
rNAPc2	N/A	Not available	NHP: 33% survival with treatment 24 h post-exposure; delayed time to death [25]	N/A	Protein; inhibits tissue factor/factor VIIa complex
Convalescent whole blood/plasma	Whole virus/glycoprotein	N/A	NHP: no efficacy with whole blood [59]; efficacy with concentrated IgG from survivors [60]	N/A	
AVI-6002	VP24/VP35	Not available	NHP: up to 63% survival at 40 mg/kg [61–63]	N/A	Combination of AVI-7537 and AVI-7539 phosphorodiamidate morpholino oligomers
AVI-7537	VP24	0.585 μM [61]	NHP: 40 mg/kg; 75% survival [63]	N/A	Phosphorodiamidate morpholino oligomer

*BID* twice daily, *PO* oral, *GP* guinea pig, *IM* intramuscular, *IP* intraperitoneal, *IV* intravenous, *QD* once daily, *C*_*ss*_ steady state concentration, *C*_*max*_ maximum concentration, *T*_*max*_ time for peak plasma concentration, *t*_*1/2*_ plasma half-life, *PPB* plasma protein binding

As a variety of assays have been used to assess the in vitro efficacy of compounds against EBOV, it can be difficult to compare results across different platforms and techniques. This is readily apparent in instances where a compound tested in parallel against both pseudotyped and wild-type virus generates different EC_50_/IC_50_ values for each assay. The discrepancy may be due to differences in GP expression between the two types of viral particles [21].

In addition to the screening of novel compound libraries and lead optimization campaigns, marketed drugs originally intended for other indications such as cancer, depression, malaria and bacterial infections have also been screened and identified as having anti-EBOV activity. These include toremiphene, clomipheme, amodiaquine, azithromycin, and chloroquine (Table 1), most of which have been classified as cationic amphiphilic drugs (CADs) [32•]. However, the mechanism of action of some of these drugs against EBOV is unclear. Screening on behalf of drug repurposing efforts has also identified hits against other viral targets such as VP24 [64].

### Pharmacokinetics and Tolerability of EBOV Therapeutics (*Adequate Drug Exposure at the Site of Action)*

Table 1 includes PK and tolerability data, both of which are critical for the selection of compounds capable of providing in vivo efficacy. Drug tolerability is especially important for preclinical EBOV therapeutic studies since survival is viewed as a key efficacy endpoint. As a rule of thumb, free drug concentrations at the target site should be sustained above the EBOV EC_50_ values when administered at a dose below the maximum tolerated dose (MTD). This brings up a key question of how the “target site” is defined. Researchers typically compare plasma PK curves (converted to unbound plasma concentrations) to the corresponding EBOV EC_50_ (or EC_90_) value to design a dosing regimen and/or determine if a compound is capable of providing enough exposure to merit efficacy testing. However, EBOV is present in macrophages, dendritic cells, and monocytes by Day 2 post-infection, and in fibroblasts, Kupffer cells, polymorphonuclear cells, endothelial cells, adrenal cortical cells, tubular epithelium, stromal cells, stromal stellate cells, tonsillar epithelium, and hepatocytes—and practically every tissue—by Day 5 [65, 66]. Moreover, EBOV has been found to persist in the semen and eyes of EBOV survivors [67]. Therefore, effective EBOV therapeutics should not be exquisitely selective for any particular cell or tissue, but rather may need to be well-distributed (e.g., high volume of distribution, *V*_*d*_). Researchers with drugs at early stages of development often face the dilemma of whether to use plasma alone to assess adequate drug exposure, or to also invest in more rigorous PK studies that involve defining the cell/tissue distribution of the drug. This is even more critical for nucleosides since the plasma half-life of the nucleoside is often very short due to rapid permeation into cells followed by intracellular conversion to their corresponding nucleoside triphosphate (TP, the active drug) where they can persist (longer half-life). For example, in the mouse BCX4430 has a 10 min half-life in plasma, but its TP in liver has a 4.3 h half-life. This conversion was found to be greater in mouse hepatocytes compared to human (Table 1), highlighting the importance of interspecies translation. Noteworthy is that the efficacious dose in the mouse (150 mg/kg, IM) corresponded to liver TP levels that were ×2.5 above the EBOV EC_50_. For another nucleoside, GS-5734, an effective dose that achieved 100% protection in NHPs (10 mg/kg) corresponded with rapid uptake into monkey PBMCs and triphosphate levels persisting above the EBOV EC_50_ over a 24-h period.

For many of the drug repurposing efforts, the maximum unbound drug concentrations in human plasma at the highest FDA-approved doses are well below the EBOV EC_50_ values (e.g., CADs in Table 1). Where mouse PK data are available (chloroquine, toremiphene, azithromycin, sertraline), unbound plasma levels appear insufficient, with the exception of azithromycin (50 mg/kg, PO) where the mouse PPB is low [68]. It is surprising that no improvements to survival were observed in the mouse by oral administration of azithromycin, especially since adequate plasma and tissue exposures were likely achieved at the 100 mg/kg dose. This result suggests that the CAD mechanism is not an effective approach to treat EVD.

### Preclinical Efficacy in Animal Models (*Expression of Pharmacological Activity in the Target Tissue*)

Following the identification of active leads and subsequent determination of their PK and tolerability, promising therapeutic compounds are advanced into preclinical studies focused on gathering data that will inform future clinical trial design and development of a promising therapeutic candidate into a safe and marketable product. This includes evaluation in animal models of the disease or condition to assess in vivo efficacy. Therapeutics in development for the treatment of EVD are tested in lethal EBOV models in mice, guinea pigs, and/or nonhuman primates (NHPs). Due to the cost, lower risk, and relative ease associated with rodent and small animal models, many therapeutics for EBOV are initially evaluated in mice and/or guinea pigs. However, wild-type EBOV is not lethal in adult immunocompetent mice or guinea pigs; both models require the use of adapted virus generated through repeated serial passage. Both models also lack some of the salient features of EVD in humans, including alterations in immune cell populations for guinea pigs and hemorrhagic and coagulation abnormalities for mice. Efficacy in both the rodent and guinea pig models is measured in terms of survival, with reduction in weight loss and extended time to death serving as alternative endpoints. Rodent models are also particularly useful for testing and validating novel targets and mechanisms of action prior to performing more intensive NHP studies. For example, target validation studies reveal that viral replication and mortality in the mouse model are decreased following VP35 inhibition [69].

Candidates that demonstrate efficacy in the mouse or guinea pig models may advance into the NHP model, which is considered to be the most accurate surrogate for human EVD due to the fact that the clinical picture in NHPs is remarkably similar to humans in terms of hemorrhagic manifestations, coagulopathy, and pathology [70]. Efficacy in the NHP model is measured primarily by survival; however, reduction in viral load and delayed time to death have also been used. For example, treatment with interferon-β prolongs survival in EBOV-infected macaques [24]. However, efficacy in the rodent model does not always translate to the NHP model. This may be due in part to the fact that EBOV does not cause the same disease manifestations in mice as in NHPs. Alternatively, the threshold for achieving protection in a mouse may be lower than that in NHPs.

The choice of animal model is occasionally dependent on the limitations associated with the different species. For example, some host-modulating drugs cannot be evaluated in rodent models as the target may not be expressed in mice. Alternatively, some drugs may possess properties that render them unsuitable for evaluation in an animal model. For example, evaluation of brincidofovir in the NHP model can be challenging due to metabolism of the drug in primates. The conversion of brincidofovir to its active form is significantly less efficient in NHPs than other species including mice and humans, resulting in lower systemic exposure [71]. As such, efficacy data for brincidofovir will need to be generated from human clinical studies. Other host-modulating drugs are dependent on the inflammatory response for which the translation from mice to human can be questioned [72]. Table 1 includes relevant in vivo efficacy data for various EBOV therapeutics under development.

### Clinical Trials and Observational Studies of EBOV Therapeutics

The 2014–2016 outbreak in West Africa motivated the initiation of multiple clinical trials for lead candidates that previously demonstrated efficacy against EBOV in animal models. Clinical trials are divided into three phases I, II, and III, which differ in their objectives and scope. The EBOV therapeutics in advanced development have only been evaluated in Phase I and Phase II trials for safety and efficacy, and due to the severity and urgency of the disease, but limited duration of the outbreak, clinical studies carried out during the outbreak have lacked proper controls and/or statistical power. More recent EBOV drug candidates such as GS-5734 and BCX4430 have completed Phase I trials and the former is currently being tested for its ability to reduce the viral load in the semen of male EBOV survivors (**Table** 2). AVI-6002 and AVI-7537 have completed Phase I trials but are not being developed by manufacturer despite preclinical efficacy data in three species and encouraging human data for safety, tolerability and PK.

**Table 2:** Human clinical data

Compound/drug	EBOV clinical trial phase	Results/status	Other clinical data
BCX4430	Phase I (NCT02319772)	Phase I complete; results not available yet	N/A
Brincidofovir	Phase II (NCT02271347)	Terminated due to low enrollment; not currently under further development as EBOV therapeutic [73]	Administered to 5 patients during the outbreak, often in combination with other therapies [52, 74]
GS-5734	Phase I	Phase I complete; Phase II for efficacy in survivors with viral persistence in semen (NCT02818582)	Administered to a newborn in combination with ZMapp and buffy coat transfusion; patient survived [75]
TKM-100802	Phase I (NCT02041715)	Terminated	100802 administered to two patients in combination with convalescent plasma; both survived [76]
TKM-130803	Phase II (PACTR201501000997429)	Terminated early; did not demonstrate efficacy [77]; development has been suspended	
Favipiravir (T-705)	Phase II (NCT02329054: JIKI; NCT02662855: Sierra Leone)	Efficacy in patients with low to moderate levels of virus (Ct values >20) [78]	Administered with ZMab to a patient who recovered [79]; administered to a patient with convalescent plasma who recovered [80]; retrospective study indicated increased survival and lower viral loads [81]
ZMapp	Phase II (NCT02363322)	Inconclusive efficacy due to insufficient statistical power [82••]	Administered to patients during the outbreak, often in combination with other therapies
AVI-6002/ AVI-7537	Phase I (AVI-6002: NCT01353027; AVI-7537: NCT01593072)	6002: Favorable safety and tolerability 7537: Terminated prior to enrollment; further development has been suspended	N/A
IFN-β	Phase I/II (ISRCTN17414946)	Results not yet released	N/A
Amiodarone	Phase II (NCT02307591)	Terminated early; reduction in case-fatality rate; not statistically significant	–
Convalescent plasma/blood	Phase I/II: NCT02333578 Phase II/III (NCT02342171; ISRCTN13990511)	Completed; results from one study found no improvement in efficacy in treated group [83]	Whole blood: 1995 Kikwit outbreak—7 out of 8 survivors [84]; administered to patients during the outbreak, often in combination with other therapies
FX-06	N/A	Not under current investigation for EBOV indication	2014: 3-day treatment course (400 mg/kg loading dose +200 mg/kg maintenance dose) was administered to a patient in combination with self-administration of amiodarone and intermittent treatment with favipiravir; patient survived [27]

Although many of the therapeutic options highlighted in the 2014–2016 outbreak had favorable Phase I outcomes, safety concerns have been identified for some products. One example is TKM-100802 which was evaluated in a Phase I trial in healthy individuals in January 2014. The trial was put on hold by the FDA due to safety concerns arising from the development of flu-like symptoms in treated individuals which was ultimately linked to cytokine release triggered by the action of the siRNA [85]. The hold was initially relaxed to allow for expanded access for EVD patients during the outbreak and was ultimately removed, enabling the study to resume at a lower dose than the one initially tested [85].

Phase II/III trials are designed to assess efficacy, with the gold standard being the randomized controlled trial in which patients are randomized to placebo or treatment arms. Although reduction in mortality is the most sought after endpoint in these trials, secondary endpoints such as reduction in viral loads are also measured. The objectives for the trial are outlined prior to its initiation; if early analysis of the trial data indicates that the objectives are not likely to be achieved, the trial may be terminated early on the basis of “futility.” Following successful restart and conclusion of its Phase I trial, TKM-130803 entered a single-arm Phase II trial in March 2015 in Sierra Leone [77]. The study was prematurely terminated due to the lack of efficacy data. Further development of TKM-Ebola has been suspended by the manufacturer (Tekmira press release 2015).

In order to demonstrate a statistically significant benefit for the therapeutic, clinical trials must be appropriately powered, requiring a minimum number of participants. Enrolling sufficient numbers of clinical trial participants is often challenging and may contribute to the success or failure of a study. Low enrollment can be due to factors such as cultural ideologies, inaccessibility of trial sites, and communication challenges. The PREVAIL II study, which evaluated ZMapp in a randomized controlled trial in Liberia, Guinea, Sierra Leone, and the USA, did not produce any statistically definitive conclusions concerning the efficacy of the treatment due to low enrollment numbers [82••]. Similarly, the Phase II trial for brincidofovir to assess safety, tolerability and efficacy was discontinued after 1 month due to a lack of enrollment [77]. Brincidofovir is not currently being advanced as an EBOV therapeutic candidate.

Statistical power is not the only key characteristic to a well-designed study. Favipiravir was tested for efficacy in 2014 in the JIKI trial (a multicenter proof-of-concept non-comparative trial conducted in four Ebola treatment centers in Guinea) which was heavily critiqued for its design: it was not randomized and relied on the use of historical controls [78]. Due to this, many found the results of the study to be difficult to reliably interpret. The current efficacy data for favipiravir is inconclusive and suggests that the drug may only be effective in treating patients with low to moderate levels of virus (CT values > than 20) [78]. Similarly, convalescent plasma was evaluated in a non-randomized, historically controlled Ebola-Tx trial in Guinea; it did not appear to demonstrate an improvement in survival [83]. The failure of some EBOV drugs to demonstrate efficacy in Phase II trials, as well as the occasional challenges in interpreting such clinical data, highlights the importance of well-designed studies. However, design of these trials may present ethical issues that are associated with high mortality pathogens such as EBOV. Randomized, controlled studies typically randomize participants to either an untreated or placebo control arm or a treated arm. However, it is considered unethical to provide no treatment for a high mortality pathogen such as EBOV. To remedy this concern, many studies have used supportive care as the control arm in an effort to assess whether the therapeutic under evaluation improves survival as compared to the current standard of care. Many of the drugs evaluated for efficacy in clinical trials have also been administered to EBOV-infected individuals under expanded access or emergency use authorization. In such cases, investigational drugs or drugs approved for other indications may be administered to critical patients. Several patients received brincidofovir during the outbreak under such circumstances, including one who received brincidofovir in combination with convalescent plasma and supportive care and survived [74]. ZMapp was also administered on a compassionate use basis to EVD patients, including several healthcare workers who survived. However, none of these treatments appeared to demonstrate statistically powered efficacy against EBOV. Part of the difficulty in interpreting such data is due to the fact that these patients often received more than one experimental treatment in combination. In such cases, it can be difficult to ascertain which drug was responsible for the therapeutic effect.

### Challenges for EBOV Drug Discovery and Development

Although many options for the treatment of EVD have begun to enter the clinical trial pipeline, there is still no FDA-approved therapy. The cessation of the 2014–2016 outbreak has returned EBOV to its previous status as the causative agent of limited, sporadic outbreaks. Due to this, as well as the unpredictability of such outbreaks, Phase II/III clinical trials aimed at assessing efficacy are difficult at the present time. While it still remains possible to evaluate safety, dose ranges, and adverse events in Phase I trials, many early trials are typically conducted at research sites in the USA. However, the populations in which safety is initially evaluated are often distinct from those in which outbreaks occur. Due to genetic and immunological differences among different patient populations, care should be taken when interpreting Phase I safety data prior to collection of additional safety data in diverse populations in Phase III. In addition, the patient populations within the historic outbreak regions are susceptible to malaria which may present similar triggers for treatment (e.g., fever, headache, muscle pain, fatigue, diarrhea, vomiting), and anti-malarial co-medication may give rise to drug-drug interactions and potentially confound phase II/III results.

The use of the FDA Animal Rule as a potential mechanism for drug approval has been proposed for EBOV. The Animal Rule was developed for high-consequence pathogens for which efficacy studies or field trials in humans are not feasible or ethical. Approval under the Animal Rule requires that efficacy must be demonstrated in at least one animal model that accurately reflects human disease. Additionally, the mechanism of action of the drug must be characterized. Occasionally drugs have shown efficacy against EBOV without a true identified target or mechanism of action. One prominent example is brincidofovir which was investigated as a therapeutic for adenovirus, smallpox, and cytomegalovirus [86]. As its mechanism of action is against dsDNA viruses, it is currently unknown how the compound inhibits an RNA virus such as EBOV.

Some of the therapeutics shown to be efficacious present challenges for large-scale production and formulation. The antibodies that make up the ZMapp cocktail are manufactured in tobacco plants (*Nicotiana benthamiana*). As such, there are limitations to manufacturing the antibodies, with supplies of the drug an issue during the outbreak. To remedy this problem, the manufacturing of ZMapp in Chinese hamster ovary (CHO) cells, which has been successfully utilized for other antibodies, has been developed and investigated [87]. An ideal therapeutic candidate for EBOV can be rapidly synthesized in large quantities so it can be made readily available during an outbreak. Convalescent plasma/serum can be similarly difficult to obtain large quantities of as it requires that samples be obtained from infected individuals.

The sheer magnitude and speed of the 2014–2016 outbreak, coupled with the challenges with the drug discovery and development process, necessitated the exploration of alternative strategies. One of these options was the repurposing of drugs in an effort to accelerate the process to place an approved therapy at the disposal of those in need more quickly. Drug repurposing, or the investigation of approved drugs or compounds for new indications, has been a hotly contested issue in the field. The primary advantage of using repurposed drugs is that they have been previously approved by the FDA for other indications. FDA approval is accompanied by a known safety profile, including toxicity, pharmacokinetic, pharmacodynamics and dosing data. As such, repurposed drugs may be accelerated through the FDA approval process by bypassing Phase I, expediting the process by months to years. The use of repurposed drugs may also alleviate some of the logistical considerations and challenges with development of a new drug, including manufacturing and distribution. However, some critics of drug repurposing argue that while such efforts may cut down on the development timeline, any unfavorable data gained from additional clinical trials may be detrimental to the current approval status for these drugs. Additionally, it may be difficult to discern the mechanism of action against EBOV for these agents. Unlike direct antiviral targets or targets within the host that are known to be critical to the virus life cycle or immune response, many of these repurposed drugs target processes that on the surface are seemingly unrelated to EBOV infection. Experiments aimed at identifying the mechanism of action of these compounds may provide new insights into the EBOV life cycle.

Additional data that has emerged from the 2014–2016 outbreak has indicated that EBOV may persist in bodily fluids such as semen for months following recovery from infection [88, 89]. In some instances, virus persistence in the semen has been implicated in the sexual transmission of EBOV from survivors to their partners [90]. As such, the development of therapeutics that are capable of eliminating EBOV from bodily fluids has become a topic of investigation. The PREVAIL IV trial is currently underway to investigate the ability of GS-5734 to eliminate persistent viral RNA in semen of survivors (NCT02818582).

## Conclusions

The drug discovery pipeline is a challenging and time-consuming process that is often plagued by high costs and high attrition rates, with very few of the numerous compounds evaluated in the early discovery stages ever making it to the clinic. The vast majority of the drug discovery efforts for Ebola, including those discussed here, have focused on EBOV Zaire. However, there is considerable interest in developing a therapeutic with broad-spectrum activity against other filoviruses such as Marburg virus and Sudan virus, or other viral pathogens. Some of the therapeutics currently under evaluation, such as GS-5734 with activity against both EBOV and MARV and BCX4430 with activity against multiple RNA viruses, are promising candidates [11]. Importantly, these drugs have demonstrated adequate triphosphate exposure at safe doses and satisfy the fundamental principles of PK/PD relationships. Further investigation utilizing well-designed and statistically powered clinical studies should be considered. However, in the absence of a current outbreak, alternative strategies including licensure through the Animal Rule may be necessary.
